# Heterosubtypic Antiviral Activity of Hemagglutinin-Specific Antibodies Induced by Intranasal Immunization with Inactivated Influenza Viruses in Mice

**DOI:** 10.1371/journal.pone.0071534

**Published:** 2013-08-16

**Authors:** Mieko Muramatsu, Reiko Yoshida, Hiroko Miyamoto, Daisuke Tomabechi, Masahiro Kajihara, Junki Maruyama, Takashi Kimura, Rashid Manzoor, Kimihito Ito, Ayato Takada

**Affiliations:** 1 Division of Global Epidemiology, Research Center for Zoonosis Control, Hokkaido University, Sapporo, Japan; 2 Division of Molecular Pathobiology, Research Center for Zoonosis Control, Hokkaido University, Sapporo, Japan; 3 Division of Bioinformatics, Research Center for Zoonosis Control, Hokkaido University, Sapporo, Japan; Public Health Agency of Canada, Canada

## Abstract

Influenza A virus subtypes are classified on the basis of the antigenicity of their envelope glycoproteins, hemagglutinin (HA; H1–H17) and neuraminidase. Since HA-specific neutralizing antibodies are predominantly specific for a single HA subtype, the contribution of antibodies to the heterosubtypic immunity is not fully understood. In this study, mice were immunized intranasally or subcutaneously with viruses having the H1, H3, H5, H7, H9, or H13 HA subtype, and cross-reactivities of induced IgG and IgA antibodies to recombinant HAs of the H1–H16 subtypes were analyzed. We found that both subcutaneous and intranasal immunizations induced antibody responses to multiple HAs of different subtypes, whereas IgA was not detected remarkably in mice immunized subcutaneously. Using serum, nasal wash, and trachea-lung wash samples of H9 virus-immunized mice, neutralizing activities of cross-reactive antibodies were then evaluated by plaque-reduction assays. As expected, no heterosubtypic neutralizing activity was detected by a standard neutralization test in which viruses were mixed with antibodies prior to inoculation into cultured cells. Interestingly, however, a remarkable reduction of plaque formation and extracellular release of the H12 virus, which was bound by the H9-induced cross-reactive antibodies, was observed when infected cells were subsequently cultured with the samples containing HA-specific cross-reactive IgA. This heterosubtypic plaque reduction was interfered when the samples were pretreated with anti-mouse IgA polyclonal serum. These results suggest that the majority of HA-specific cross-reactive IgG and IgA antibodies produced by immunization do not block cellular entry of viruses, but cross-reactive IgA may have the potential to inhibit viral egress from infected cells and thus to play a role in heterosubtypic immunity against influenza A viruses.

## Introduction

Influenza A viruses are divided into subtypes based on the antigenicity of two envelope glycoproteins, hemagglutinin (HA) and neuraminidase (NA). To date, H1–H16 and N1–N9 subtypes have been found in wild aquatic birds, the natural reservoir of influenza viruses [1]–[3]. It is known that HA is the major target of neutralizing antibodies against influenza viruses [4], and HA-specific antibodies are principally subtype-specific. Therefore, the currently used inactivated influenza vaccines, which rely on the induction of serum neutralizing antibodies, are not effective against viruses whose HA antigenicities are different from those of the vaccine strains [5]. On the other hand, infection with influenza A virus usually affords some protection against reinfection with viruses having different subtypes [6]. It has been believed that this heterosubtypic protection is mainly mediated by memory cytotoxic T lymphocytes (CTL) recognizing conserved epitopes of viral internal proteins presented with MHC class I on the surfaces of infected cells [7], [8]. Therefore, the contribution of virus-specific antibodies to the heterosubtypic immunity has been thought to be limited and has not been evaluated properly.

However, recent reports demonstrated the presence of HA-specific monoclonal antibodies that had cross-neutralizing activity against multiple HA subtypes of influenza A virus strains [9]–[16]. Biological and structural analyses indicated that these antibodies had the potential for either of the known neutralization mechanisms, preventing viral attachment to host cells or conformational change/proteolytic cleavage of HA, both of which are essential for virus entry into host cells. Although it may be difficult to induce high levels of cross-neutralizing antibodies since these antibodies are thought to recognize minor epitopes, recent studies have suggested that such antibodies are indeed produced in some individuals [17], 18.

On the other hand, it was reported that heterosubtypic immunity was induced by intranasal immunization of mice with formalin-inactivated influenza A viruses, whereas subcutaneous immunization only protected mice from homologous viruses [6], [19], [20]. Interestingly, this cross-protection was dependent on B cell, but not on CTL activity [19]. However, in vitro neutralizing activity of antibodies was not detected in the sera and respiratory secretions of immunized mice. Taken together, these studies led to the hypothesis that HA-specific antibodies, including nonneutralizing antibodies, also play important roles in heterosubtypic immunity against influenza A viruses.

In this study, we found that subcutaneous and intranasal immunization of mice with inactivated viruses induced IgG and/or IgA antibodies that bound to HAs of multiple subtypes, whereas IgA antibodies were not detected remarkably in mice immunized subcutaneously. By a standard plaque-reduction neutralization test in which viruses were mixed with antibodies prior to inoculation into cultured cells, the neutralizing activity was detected only against the homologous virus (i.e., the same subtype as the immunogen). Interestingly, however, when cells infected with viruses were subsequently maintained in the presence of IgA (but not IgG) antibodies, reduced plaque formation of viruses with heterologous subtypes was observed. Here we discuss a possible role of cross-reactive nonneutralizing IgA antibodies in the heterosubtypic immunity against influenza A viruses.

## Materials and Methods

### Viruses and Cells

Influenza A virus strains, A/Puerto Rico/8/1934 (H1N1), A/Adachi/2/1957 (H2N2), A/Aichi/2/1968 (H3N2), A/duck/Czechoslovakia/1956 (H4N6), A/rg Viet Nam ΔHA/1194/2004 (H5N1) [16], A/shearwater/Australia/1/1972 (H6N5), A/seal/Massachusetts/1/1980 (H7N7), A/turkey/Ontario/6118/1968 (H8N4), A/Hong Kong/1073/1999 (H9N2), A/chicken/Germany/N/1949 (H10N7), A/duck/England/1/1956 (H11N6), A/duck/Alberta/60/1976 (H12N5), A/gull/Maryland/704/1977 (H13N6), A/mallard/Astrakhan/263/1982 (H14N5), A/duck/Australia/341/1983 (H15N8), and A/black-headed gull/Sweden/5/1999 (H16N3) were kindly provided by Dr. H. Kida, Graduate School of Veterinary Medicine, Hokkaido University, Sapporo, Japan and used for immunization of mice, construction of plasmid expressing recombinant HAs, and plaque-reduction assays using Madin-Darby canine kidney (MDCK) cells. MDCK cells were maintained in Eagle’s minimal essential medium (MEM) (GIBCO) supplemented with 10% calf serum. Human embryonic kidney (HEK) 293T cells were maintained in Dulbecco’s modified Eagle’s medium supplemented with 10% fetal calf serum.

### Immunogens

Virus strains of H1, H3, H5, H7, H9, and H13 HA subtypes were propagated in the allantoic cavity in 10 day-old embryonated chicken eggs at 35°C for 48 hours, then the infectious allantoic fluid was collected. Virus particles were concentrated and purified by high-speed centrifugation of the allantoic fluid passed through a 10–50% sucrose density gradient. Purified viruses were resuspended in phosphate-buffered saline (PBS). The protein concentration of each purified virus was measured based on the optical density at 280 nm. All protein concentrations were standardized for each immunogen as relative to each other based on OD280 values. The purified viruses were treated with 0.3% formalin at the concentration of 20 mg viral proteins per 1 ml at 4°C for one week. Inactivation of these viruses was confirmed by the absence of detectable hemagglutination activity following inoculation of the treated materials into embryonated eggs. Each inactivated virus was diluted to adequate concentrations with PBS before immunization of mice.

### Immunization and Sample Collection

Six-week-old female BALB/c mice (Japan SLC, Inc.) were inoculated intranasally with inactivated viruses (100 µ5;g in 50 µl) or 50 µl of PBS under anesthesia with isoflurane, or injected subcutaneously with inactivated viruses (100 µg in 200 µl) or 200 µl of PBS. Five or ten mice were used for each group. Mice were immunized three times at three-week intervals. One week after the last immunization, mice were euthanized with isoflurane and serum, trachea-lung wash (TW), and nasal wash (NW) samples were collected as described previously [21]. These samples were stored at −80°C until use. Samples from each group were pooled in equal volumes and used for the antibody assays described below. For plaque-reduction neutralization tests, serum samples were pretreated with receptor destroying enzyme, RDE (II) “SEIKEN” (Denka Seiken Co., Ltd., Japan) according to the manufacturer’s instructions. The TW and NW samples were also pretreated with one-tenth volume of RDE. Animal studies were carried out in strict accordance with the Guidelines for Proper Conduct of Animal Experiments of the Science Council of Japan [22]. The protocol was approved by the Hokkaido University Animal Care and Use Committee. Three independent experiments were conducted using 5 or 10 mice/each group/experiments for immunization with H9N2, and in each of these experiment the serum antibodies obtained from mice showed the same spectrum of cross-binding ability to HAs (data not shown).

### Expression of Recombinant HA and NA

Viral RNAs were extracted using a QIAamp Viral RNA Mini Kit (Qiagen). After reverse transcription with Moloney murine leukaemia virus reverse transcriptase (Invitrogen) using Uni12 primer (5′-AGCAAAAGCAGG), HA and NA genes were amplified by PCR using gene-specific primer sets [23]. PCR products were purified with a Wizard SV Gel PCR Clean-up system (Promega) and cloned into pCAGGS, the mammalian expression plasmid. HEK293T cells (2×10^6^) were plated on 10 cm dish, and 24 hours later, cells were transfected with pCAGGS expressing the recombinant HA or NA of each subtype using TransIT^®^-LT1 transfection reagent (Minus). At 48 hours after transfection, the recombinant HAs or NAs were extracted using a eukaryotic membrane protein extraction reagent kit (Thermo Fisher Scientific), according to the manufacturer’s protocol. The extracted membrane proteins were appropriately diluted (1∶2000–8000) with PBS to give the highest optical density (O.D.) values at 450 nm for antisera or monoclonal antibodies (repository of our laboratory) specific to the respective HA or NA subtypes and used as antigens for enzyme-linked immunosorbent assay (ELISA).

### ELISA

IgG and IgA antibodies in the serum, TW, and NW samples were measured by ELISA as described previously [24]. Briefly, ELISA plates (Nunc Maxisorp) were coated with the HA (H1–H16) or NA (N2 and N5) antigens, and washed with PBS containing 0.05% Tween 20 (PBST), followed by blocking with 3% skim milk in PBS. Serum, TW, and NW samples were diluted at 1∶100, 1∶3.5, and 1∶3.5, respectively, in PBST containing 1% skim milk. The bound antibodies were detected using goat anti-mouse IgA (α) and goat anti-mouse IgG (γ) antibodies conjugated to horseradish peroxidase (Kirkegaard & Perry Laboratories, Inc.) diluted in PBST containing 1% skim milk. The reaction was visualized by adding 3,3′,5,5′-tetramethylbenzidine (TMB, Sigma-Aldrich) and the O.D. at 450 nm was measured.

### NA Inhibition (NI) Assay

Serum NI activities of H9N2 virus-immunized mice were measured by a standard colorimetric assay using fetuin (Calbiochem) as substrate [25]. The absorbance was measured at 549 nm. Endpoint NI titers were determined as the reciprocal of the highest serum dilution causing 50% inhibition of neuraminidase activity given in the absence of the serum. NI assay was conducted for H9N2, H12N5, and H3N2 viruses.

### Standard Plaque-reduction Test

The inhibition of viral entry into cells was evaluated by the standard procedure of plaque-reduction neutralization tests using MDCK cells. Serial dilutions of the samples (50 µl) were mixed with an equal volume of diluted virus solution (approximately 100 plaque-forming units), and incubated for 1 hour at room temperature. Then the mixture was inoculated onto a monolayer of MDCK cells on a 12-well tissue culture plate. After 1-hour incubation at 35°C, the inoculum was aspirated and cells were washed once with serum-free MEM and overlaid with MEM containing 1% Bacto-agar and trypsin (5 µg/ml) (GIBCO). The plaques were enumerated after incubation at 35°C for 2 days.

### Modified Plaque-reduction Test

To further test the potential of antibodies to inhibit virus replication, plaque-reduction tests were modified. MDCK cells cultured in 12-well plates were first inoculated with a virus solution (50–100 plaque-forming units/well), followed by incubation for 1 hour at 35°C. The inoculum was replaced with overlay medium (MEM with 1% Bacto-agar) containing the serum, TW, or NW samples at final dilutions of 1∶200, 1∶5, or 1∶5, respectively. A higher concentration (15 µg/ml) of trypsin was used for the medium containing serum samples. After incubation for 48 hours at 35°C, the overlay medium was removed, and the cells were washed 2 times with PBS and fixed with methanol. Plaques were stained with chicken antisera to the respective HA subtypes (kindly provided by Dr. H. Kida), horseradish peroxidase-conjugated rabbit anti-chicken IgY (IgG) (H+L) (Jackson Immuno Research, USA), and 3,3′-diaminobenzidine (Wako). In some experiments, to inhibit the interaction of IgA or IgG with target molecules [26], [27], goat anti-mouse IgA or IgG (polyclonal serum) (SouthernBiotech, USA) was added to the NW samples, and reacted for 1 hour at room temperature. Then, the reaction mixture or same volume of PBS was mixed with overlay medium. Anti-mouse IgA or IgG antibodies were used at 1 µg/ml (final concentration in the medium). We confirmed preliminarily that pretreatment with these anti-IgA or IgG antibodies efficiently inhibited neutralizing activities of HA-specific monoclonal IgA or IgG in vitro (data not shown). Each experiment was performed at least twice.

### SDS-PAGE and Western Blotting

MDCK cells infected with viruses at a multiplicity of infection of 1.0 were maintained with MEM containing the sample for 7 hours, and culture supernatants were collected and mixed with SDS-PAGE sample buffer. After 5–20% SDS-PAGE, separated proteins were blotted on a polyvinylidene difluoride membrane (Millipore). Chicken antisera were used as primary antibodies to detect viral proteins. The bound antibodies were detected with peroxidase-conjugated rabbit anti-chicken IgY (IgG) (H+L) (Jackson Immuno Research, USA), followed by visualization with Immobilon Western (Millipore).

### Phylogenetic Analysis

Phylogenetic analysis was based on whole amino acid sequences of HAs obtained from GenBank under accession numbers ABO21709.1 (H1), BAG72216.2 (H2), BAF48361.1 (H3), BAF48478.1 (H4), ABP51976.1 (H5), BAF36386.1 (H6), BAF02934.2 (H7), BAF43468.1 (H8), CAB95856.1 (H9), BAF46908.1 (H10), BAF43435.1 (H11), BAF43416.1 (H12), BAF46906.1 (H13), BAF43460.1 (H14), BAF48363.1 (H15), and AAV91217.1 (H16). The sequences were aligned by using GENETYX (Genetyx Corp., Japan) for Windows, version 10. A phylogenetic tree was constructed by using the neighbor-joining method in MEGA 5.1 [28].

## Results

### Both Intranasal and Subcutaneous Immunizations of Mice Induced Heterosubtypic Antibody Responses to Multiple HAs

**Figure 1:**
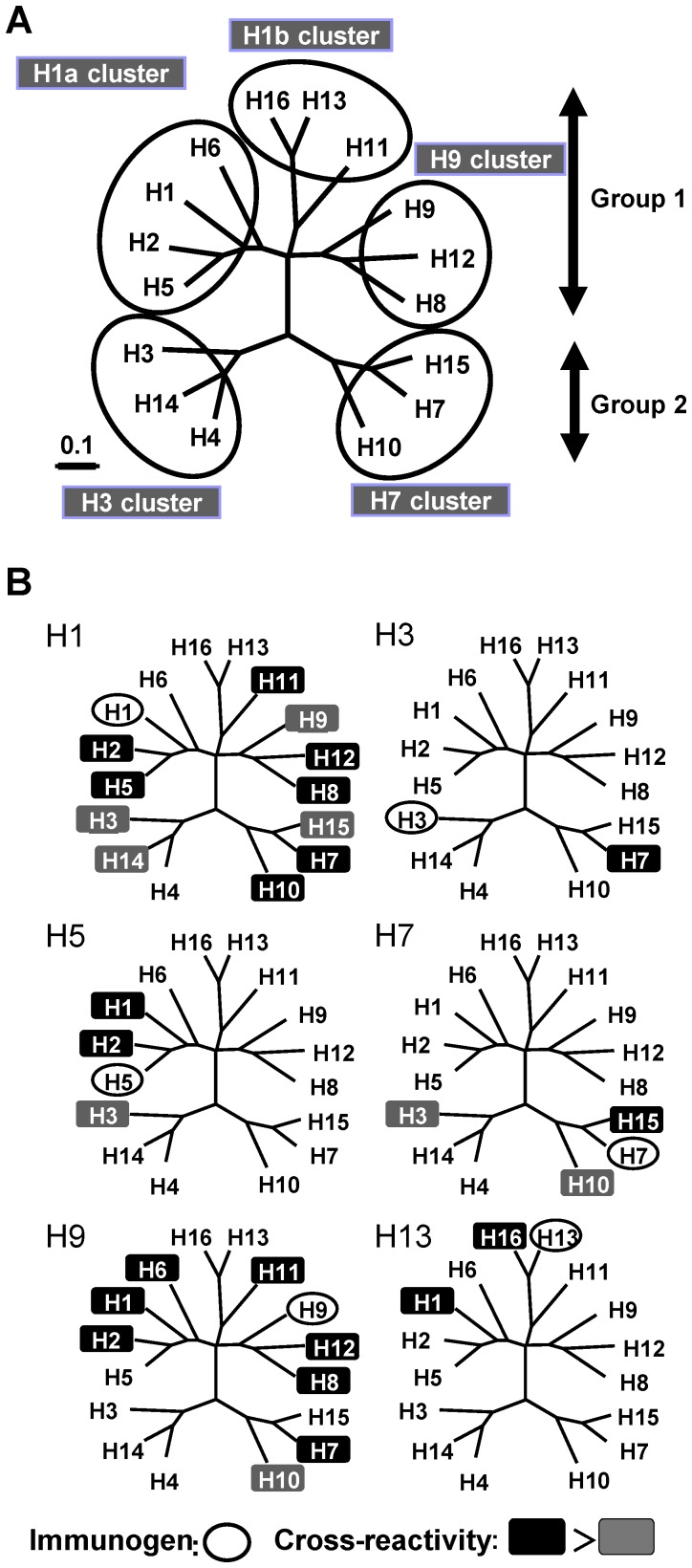
Correlations between cross-binding activities of antibodies and phylogenetic grouping of HA. (A) Phylogenetic relationships among 16 HA subtypes of influenza A viruses based on amino acid sequences. The HA amino acid sequences of the viruses used for ELISA antigens were used. (B) The subtypes of HAs to which serum IgG antibodies of subcutaneously immunized mice showed cross-binding activity (See also Figure 2). In each phylogenetic tree, the HA subtype used for immunization is circled. The HA subtypes to which antibodies showed cross-binding activity are shown in black (O.D. ≧ 1.0) or dark gray (1.0 > O.D. ≧0.5) background.

**Figure 2:**
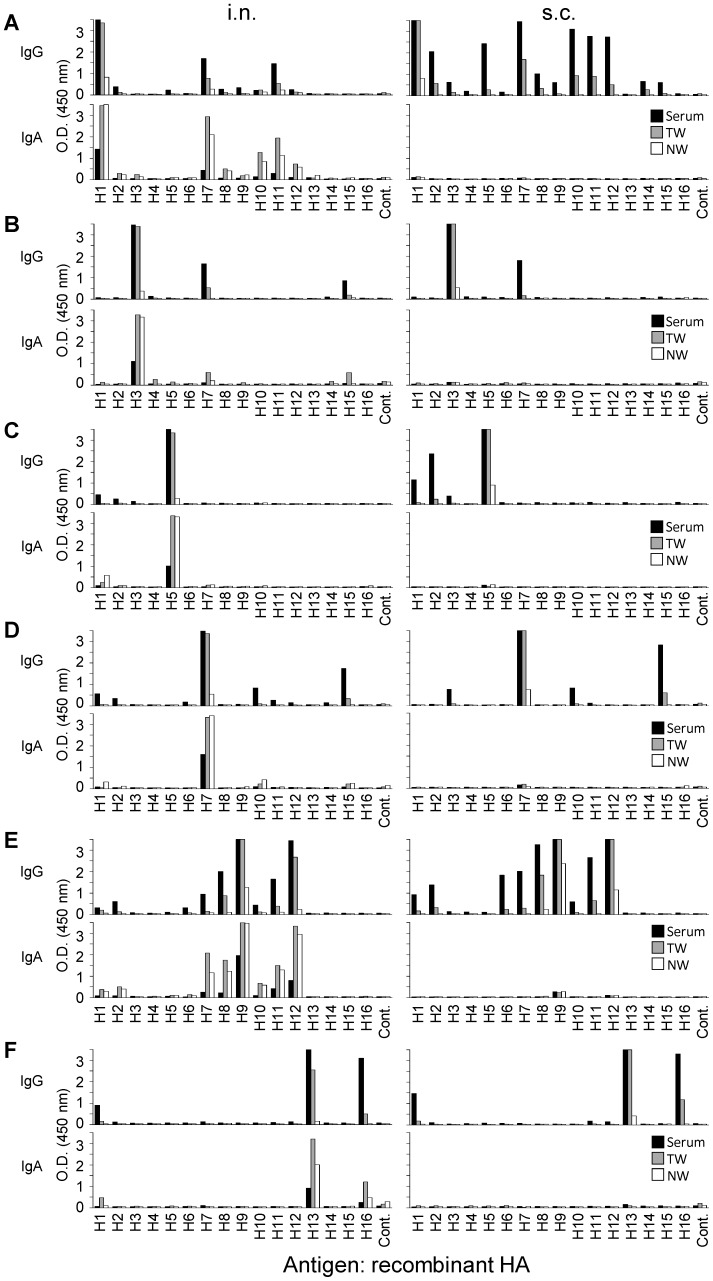
Cross-binding activities of HA-specific antibodies. Mice were immunized intranasally (i.n.) or subcutaneously (s.c.) with formalin-inactivated H1N1 (A), H3N2 (B), H5N1 (C), H7N7 (D), H9N2 (E), or H13N6 (F) viruses. Serum, TW, and NW samples were collected 7 days after the last immunization. Samples from each group were pooled and diluted at 1∶100 (serum), 1∶3.5 (TW and NW) with 1% skim milk in PBST. HA-specific IgG and IgA antibodies were detected by ELISA as described in the Materials and Methods section.

The virus strains of H1, H3, H5, H7, H9, and H13 HA subtypes were selected for immunization as representatives of each cluster in a phylogenetic tree based on HA amino acid sequences (Fig. 1A). Mice were immunized intranasally or subcutaneously with these viruses, and HA-specific IgG and IgA antibodies in serum, NW, and TW samples were analyzed by ELISA for the binding activity to HAs of H1–H16 subtypes (Fig. 2). In the serum samples of mice immunized subcutaneously with the H1N1 virus, IgG cross-reactive to H2, H3, H5, H7, H8, H9, H10, H11, H12, H14, and H15 HAs was detected (Fig. 2A, upper right). Lower levels of IgG were detected in mice immunized intranasally than in mice immunized subcutaneously (Fig. 2A, upper left). IgA was not detected remarkably in any samples of mice immunized subcutaneously (Fig. 2A, lower right). The HA subtypes to which IgG or IgA showed comparatively high cross-reactivity were almost the same (i.e., H7, H10, H11, and H12) with intranasal and subcutaneous immunizations. In intranasally immunized mice, there was no fundamental difference in the overall spectrum of cross-reactivity between IgG and IgA antibodies (Fig. 2A, upper and lower left). The spectrum of heterosubtypic responses (i.e., the HA subtypes to which induced antibodies showed cross-binding activity) varied depending on the HA subtypes of the viruses used for immunization (Fig. 2A–F); HA-specific antibodies were cross-reactive to H7 in H3N2 virus-immunized mice (Fig. 2B), to H1, H2, and H3 in H5N1 virus-immunized mice (Fig. 2C), to H1, H10, and H15 in H7N7 virus-immunized mice (Fig. 2D), to H1, H2, H6, H7, H8, H10, H11, and H12 in H9N2 virus-immunized mice (Fig. 2E), and to H1 and H16 HAs in H13N6 virus-immunized mice (Fig. 2F). As expected, there was a common observation, irrespective of the subtype used for immunization, that subcutaneous immunization induced IgG, but only slight IgA responses, whereas intranasal immunization induced both IgG and IgA responses, but IgG responses were generally lower than those of mice immunized subcutaneously.

### The Spectrum of Cross-reactive Antibodies Correlated with Phylogenetic Classification of HAs

Influenza virus HAs are phylogenetically divided into two groups (groups 1 and 2) consisting of 5 clusters (H1a, H1b, H9, H3, and H7 clusters) based on their amino acid sequences [29], [30] (Fig. 1A). On the phylogenetic tree, we mapped the HA subtypes that were recognized by cross-reactive antibodies induced by each immunogen (Fig. 1B). We found that the spectrum of the HA-specific cross-reactive antibodies was directed to particular HA subtypes that were closely related to the viruses used for immunization. However, it was noted that broader cross-reactivity beyond the clusters and groups was found for the antibodies of H1N1 and H9N2 virus-immunized mice. For example, in mice immunized subcutaneously with H9N2, the serum IgG was cross-reactive to H12 and H8 HAs, both of which belonged to the same cluster, but also to other HAs such as H6, H7, and H11 which belonged to different clusters or groups (Fig. 1B). On the other hand, it was also noteworthy that the cross-reactivity did not necessarily cover all subtypes belonging to the same clusters to which each immunogen belonged (e.g., H1-, H3-, and H13-induced antibodies did not bind to H6, H4, and H11 HAs, respectively).

### Cross-reactive Antibodies Showed no Heterosubtypic Neutralizing Activity in a Standard Plaque-reduction Test

**Figure 3:**
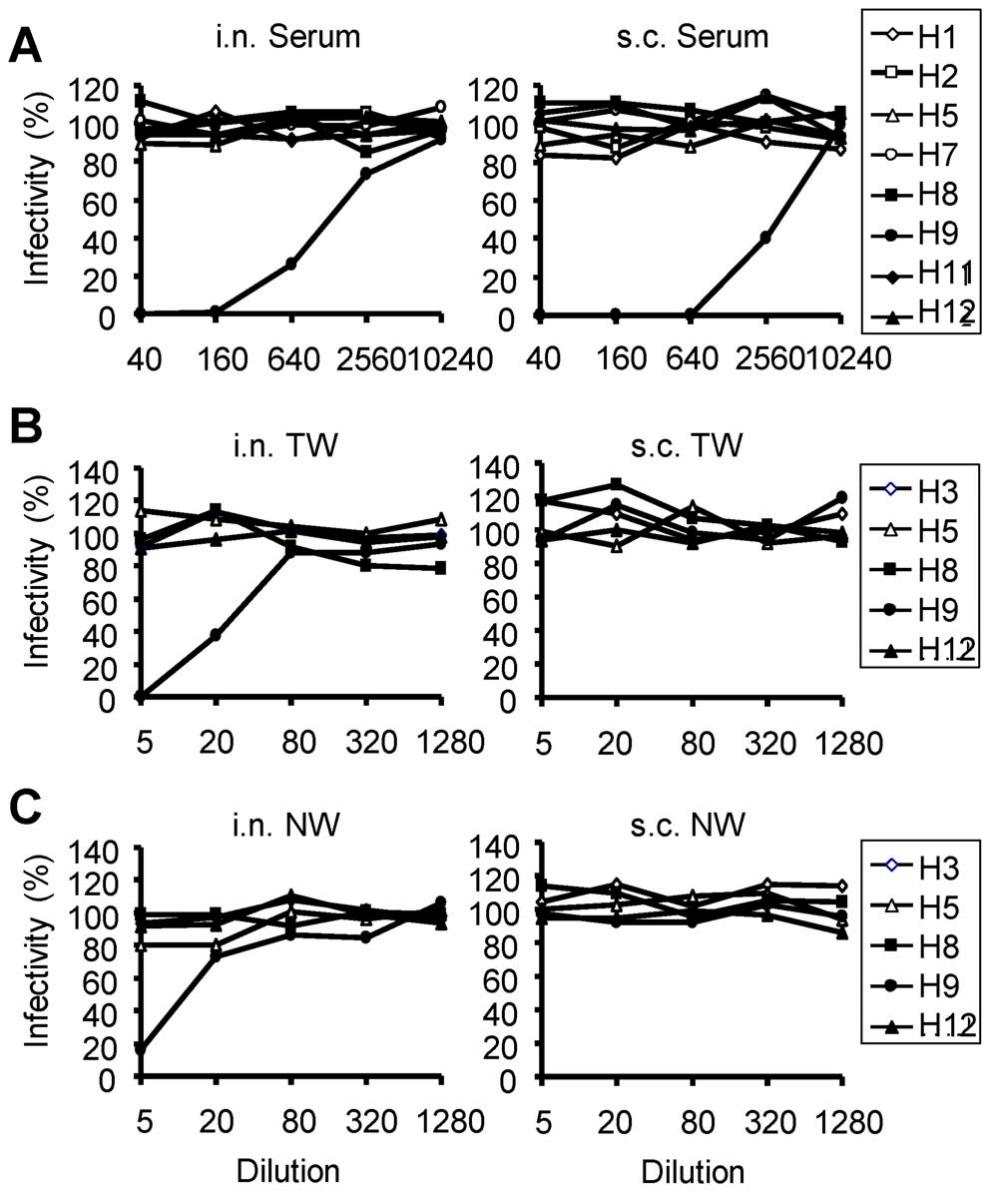
Neutralizing activities of the samples of mice immunized with the H9N2 virus in a standard plaque-reduction test. Appropriately diluted viruses were mixed at indicated dilutions with the serum (A), TW (B), or NW (C) sample of mice immunized with H9N2 intranasally (i.n.) or subcutaneously (s.c.). Neutralizing activities were evaluated by counting the number of plaques formed on MDCK cells.

To evaluate whether the induced cross-reactive antibodies had the ability to inhibit virus entry into cells (i.e., so called neutralizing activity), we focused on H9-induced antibodies that showed remarkable cross-reactivity (Fig. 2E), and a standard plaque-reduction test was carried out using the serum, TW, and NW samples of H9N2 virus-immunized mice. In this test, viruses were mixed with antibodies prior to inoculation onto MDCK cells and the reduction of infectivity was estimated by plaque counts. Viruses having H1, H2, H7, H8, H11, and H12 HAs, to which H9N2 virus-induced antibodies bound, H9N2 as a positive control, and H5N1 as a negative control to which H9N2 virus-induced antibodies are little cross-reactive in ELISA, were selected as challenge viruses. We found that the heterosubtypic neutralizing activity of antibodies was not detected in any samples even at the lowest sample dilutions tested, although the neutralizing activity against the homologous virus (i.e., H9) was clearly detected in the serum, TW and/or NW samples of immunized mice (Fig. 3A). It was noted that the TW samples of subcutaneously immunized mice did not show neutralizing activity against the homologous virus though these samples appeared to contain anti-H9 IgG comparable to the samples of intranasally immunized mice (Fig. 2E, upper).

### Plaque Formation of H12N5 Virus was Reduced in the Presence of H9N2 Virus-induced Cross-reactive Antibodies

**Figure 4:**
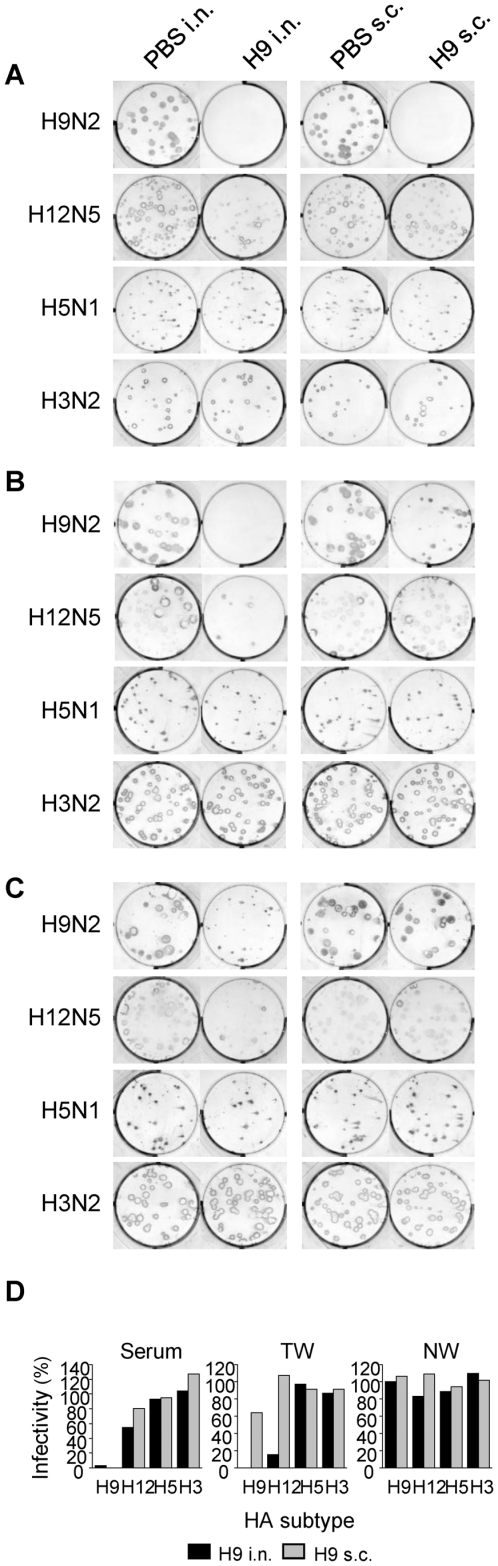
Reduced plaque formation in the presence of antibodies of mice immunized with H9N2. After being inoculated with H9N2, H12N5, H5N1, or H3N2, MDCK cells were cultured in the presence of antibodies in the serum (1∶200) (A), TW (1∶5) (B), or NW (1∶5) (C) samples of mice immunized intranasally (i.n.) or subcutaneously (s.c.). Numbers of plaques were counted and plaque reduction was calculated relative to the number of plaques formed in the presence of the samples of mice given PBS intranasally or subcutaneously for i.n. or s.c. immunized mice, respectively (D).

**Figure 5:**
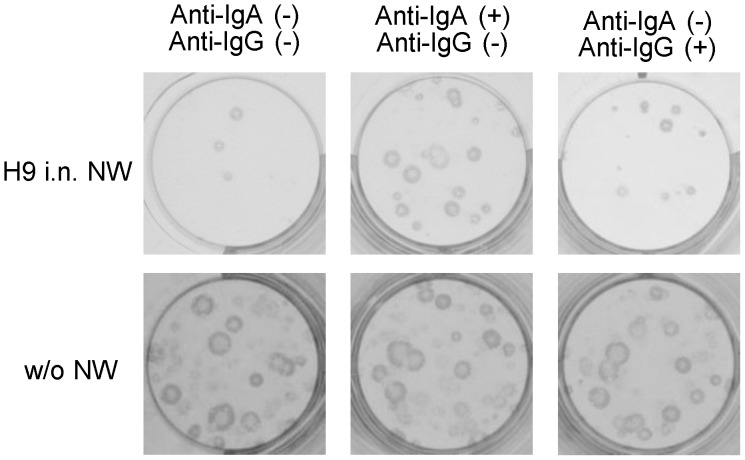
Heterosubtypic plaque reduction interfered by anti-mouse IgA antibodies. NW samples of mice immunized with H9N2 virus intranasally (i.n.) were pretreated with anti-mouse IgA or anti-mouse IgG antiserum (1 µg/ml). MDCK cells infected with H12N5 virus were incubated in the presence or absence of the NW samples treated or nontreated with the antiserum.

To further examine the antiviral activities of the cross-reactive antibodies, we modified the procedure of the plaque-reduction test. In the assay applied here, viruses were first inoculated onto MDCK cells without preincubation with antibodies and then cultured in media containing appropriately diluted samples. This assay might enable us to detect antibodies that inhibit extracellular release of virus particles from infected cells, even if antibodies do not inhibit HA-mediated entry of viruses into host cells [31], [32]. We selected viruses of 4 different subtypes as challenge viruses for this assay; H9N2 as a positive control, H12N5, which was most effectively bound by H9-induced cross-reactive antibodies in ELISA, H5N1 as a negative control to which H9N2 virus-induced antibodies are little cross-reactive, and H3N2, whose NA subtype is the same as that of the virus used for immunization (H9N2) in order to test the inhibitory effects of NA-specific antibodies. In the presence of serum antibodies of mice immunized intranasally or subcutaneously with H9N2, the homologous virus formed no visible plaques (Fig. 4A and D). Interestingly, plaque sizes and numbers of H12N5 were reduced to some extent in the presence of serum antibodies containing both IgG and IgA, of mice immunized intranasally, but the reduction was minimally found in the presence of high levels of serum IgG of mice immunized subcutaneously (Fig. 4A and D). Antibodies in the TW and NW samples exhibited more substantial differences in heterosubtypic plaque-reduction activity between intranasally and subcutaneously immunized mice. The TW and NW samples that contained cross-reactive IgG and IgA remarkably reduced the number and/or size of plaques of the H12N5 virus, whereas the samples of subcutaneously immunized mice did not show such inhibitory effects (Fig. 4B, C, and D). It was noted that the TW and NW samples of subcutaneously immunized mice contained high levels of IgG (Fig. 1E), but gave much less plaque reduction of even the homologous virus (i.e., H9) than those of intranasally immunized mice (Fig. 4B and D). Actually, the extent of plaque reduction was associated with the presence of cross-reactive anti-HA IgA in the samples. No apparent plaque reduction was observed for H3N2 and H5N1 viruses in the presence of H9N2-induced antibodies, regardless of the sample origin (i.e., serum, TW, or NW). To confirm the role of IgA antibodies in heterosubtypic plaque reduction, we pretreated the NW samples of H9N2 virus-immunized mice with goat anti-mouse IgA or anti-IgG antibodies and mixed with overlay medium in the modified plaque reduction test. As expected, the heterosubtypic plaque reduction of H12 virus was interfered with anti-mouse IgA but not anti-IgG antibodies (Fig. 5).

### Budding and Release of H12N5 from Infected Cells were Impeded in the Presence of H9N2 Virus-induced Cross-reactive Antibodies

**Figure 6:**
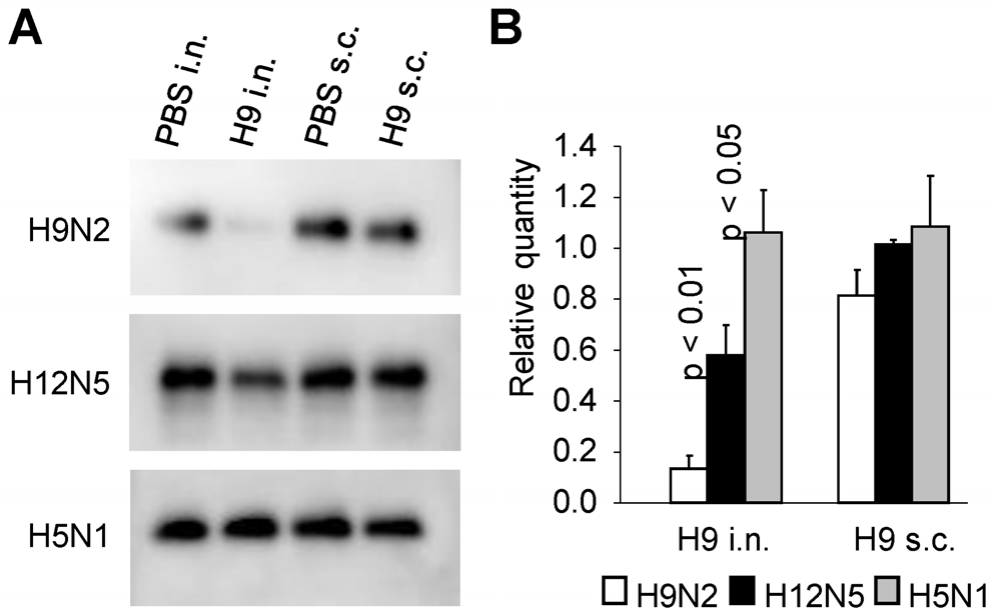
Detection of viral proteins in the supernatants of cells infected with H9N2, H12N5, or H5N1. MDCK cells were infected with viruses and cultured with the TW samples of intranasally (i.n.) or subcutaneously (s.c.) immunized mice as described in the legend of Figure 5. Supernatants were collected 7 hours after infection, and virus particles released into the supernatants was detected by Western blotting (A). The relative intensity of the HA bands compared to each of the control (PBS i.n. and s.c.) samples was obtained using Image Lab version 3.0 (BIO RAD) (B). Experiments were performed 3 times, and averages and standard deviations are shown. Student t-test was used for statistical analysis.

To gain insight into the mechanism of antibody-mediated inhibitory effects on the plaque formation, we examined extracellular release of virus particles in the presence of antibodies. MDCK cells infected with H9N2, H12N5, or H5N1 were cultured with the TW antibodies of H9N2 virus-immunized mice, and then the amounts of virus particles released into the culture supernatants were estimated by detecting viral proteins in Western blotting (Fig. 6). We found that significantly lower amounts of HAs of H9N2 and H12N5 were detected in the supernatants of infected cells cultured with TW antibodies of mice immunized intranasally with H9N2, compared with those seen in samples from subcutaneously immunized mice. No remarkable decrease of the viral particle formation was appreciable for H5N1, regardless of the immunization route. Taken together, these data suggested that cross-reactive nonneutralizing antibodies, most likely IgA, induced by H9N2 effectively inhibited the budding and release of H12N5 virus particles from infected cells.

### No Cross-reactive NI Activity was Detected in the Serum of H9N2-virus Immunized Mice

To test the possibility that the cross-reactive NI activity was correlated with the reduced viral replication and budding of the H9N2 virus, we examined NI activity in the serum samples of H9N2 virus-immunized mice against H12N5 and H3N2 viruses (Table 1). Serum of mice immunized intranasally or subcutaneously with H9N2 showed high NI titers against the homologous H9N2 virus, while little inhibition was detected against H12N5. A slight NI activity was detected in the serum of mice immunized intranasally with the H3N2 virus.

**Table 1:** Serum NI activities of H9N2 virus-immunized mice against H9N2, H12N5, and H3N2 viruses.

Virus	Immunization	NI titer
H9N2	Subcutaneous	320
	Intranasal	320
H12N5	Subcutaneous	<20
	Intranasal	<20
H3N2	Subcutaneous	<20
	Intranasal	20

## Discussion

It has been shown that intranasal immunization with an inactivated virus confers heterosubtypic protection from influenza A viruses in mice, whereas subcutaneous immunization is only effective against viruses with homologous HA subtypes [19], [20], [33], [34]. Such protection was shown to be B-cell dependent [19]. In these previous studies, cross-neutralizing antibodies were consistently undetectable in a standard neutralization test, while HA-specific antibodies with cross-binding activity were often induced [35]. Thus, this study aimed to clarify whether intranasal immunization of mice with an inactivated influenza virus generally induces HA-specific nonneutralizing antibodies reactive to multiple HA subtypes and to elucidate the potential roles of these antibodies in the heterosubtypic immunity against influenza A viruses.

We first analyzed cross-binding activities of HA-specific antibodies induced by intranasal or subcutaneous immunization with viruses having different HA subtypes (i.e., H1, H3, H5, H7, H9, or H13), and found that substantial amounts of cross-reactive IgG antibodies to multiple HA subtypes, which were predominantly related to each immunogen phylogenetically, were indeed detectable in the sera and respiratory secretions of immunized mice. There was no fundamental difference in the overall spectrum of the IgG cross-reactivity between the antibodies produced by intranasal and subcutaneous immunizations. Importantly, however, both IgA and IgG antibodies, which are the principal isotypes of mucosal immunity and systemic immunity, respectively [26], [36], were produced by intranasal immunization; whereas only the IgG antibody response was induced by subcutaneous immunization. These results suggest that the IgA antibodies may play a major role in the B-cell dependent heterosubtypic immunity induced by intranasal immunization of mice.

In a standard neutralization test, neutralizing activities of the antibodies in H9N2 virus-immunized mice were only targeted to the homologous virus, regardless of the immunization route and sample origin, which was consistent with previous studies [19], [20], [33], [34]. This results indicates that majority of the cross-reactive antibodies detected in ELISA do not recognize the so-called neutralizing epitopes that are typically located on functionally important sites of HA (e.g., the receptor binding sites). It was also noted that antibodies in the TW or NW samples of mice immunized subcutaneously did not neutralize even the homologous H9N2 virus (Fig. 3B and C), while these samples contained similar or even higher levels of IgG than in those from intranasally immunized mice (Fig. 2E). Instead, neutralizing activities of the TW and NW samples were associated with the presence of IgA, suggesting that IgA neutralized virus infectivity more effectively than IgG.

In the modified plaque-reduction test, the plaque formation of H12N5, but not H5N1 and H3N2, was suppressed in the presence of antibodies in the serum, TW, and NW samples from mice immunized with H9N2 intranasally (Fig. 4), although these antibodies did not display “classical” neutralizing activity against the H12N5 virus (Fig. 3). No plaque reduction of H12 virus was seen in the presence of serum or TW samples of negative control and H13N6 virus-immunized mice (data not shown), in which antibodies cross-reactive to H12 HA were not detected, suggesting the unlikelihood that the plaque reduction of H12 virus was due to nonspecific inhibitors in the samples. The extent of plaque reduction indeed seemed to reflect the concentration of anti-HA IgA in each sample (Fig. 2E). By contrast, no significant plaque reduction of H12N5 was observed in the samples of mice immunized with H9N2 subcutaneously. It is noteworthy that the serum, TW, and NW samples from mice immunized subcutaneously with H9N2 contained higher amounts of IgG antibodies cross-reactive to H12 HA than those of intranasally immunized mice (Fig. 2E), but did not show inhibitory activity to reduce the plaque number and size of the H12N5 virus (Fig. 4). Furthermore, the plaque reduction activity of the samples of mice immunized with H9 virus intranasally was decreased when the samples were pretreated with anti-mouse IgA antibodies but not anti-mouse IgG antibodies (Fig. 5). Taken together, these results suggested that cross-reactive IgA antibodies had the ability to reduce plaque formation, likely by preventing the budding or extracellular release of virus particles from infected cells (Fig. 6), whereas these antibodies did not block HA-mediated entry of viruses into host cells (Fig. 3).

It is known that nonneutralizing antibodies against NA or M2 protein, which are the other envelope proteins of influenza A viruses, inhibit the extracellular release of progeny viruses from host cells, and thus play a role in protective immunity [37]. These antibodies do not prevent virus entry into host cells, but significantly inhibit plaque formation of influenza viruses when infected cells are cultured in the presence of the antibodies [38], [39]. Such antibodies are thought to produce a crosslink between virus-associated NA or M2 and those expressed on the cell surface, leading to reduced budding of the virus [31], [40]–[42]. In this study, antibodies induced by immunization with H9N2 did not reduce the plaque formation of H3N2, despite having the same NA subtype. In a neuraminidase inhibition test, the serum samples of mice immunized intranasally with H9N2 showed only moderate inhibition of H3N2 but not H12N5 viruses (Table 1). Furthermore, in ELISA using recombinant NA antigens, cross-reactive antibodies to N5 were undetectable in the serum of H9N2-immunized mice (data not shown). These data suggest that NA-specific antibodies did not mainly contribute to the heterosubtypic plaque reduction observed in this study. M2-specific antibodies were also unlikely to be involved in the heterosubtypic plaque reduction seen in this study since no plaque reduction by H9-induced antibodies was observed for H3N2 and H5N1, despite the highly conserved antigenicity of the M2 protein among influenza A viruses [37]. Overall, our data suggest that some of the cross-reactive nonneutralizing anti-HA IgA antibodies induced by intranasal immunization may have similar inhibitory effects on the virus particle formation and play a role in the heterosubtypic immunity against influenza A viruses. Accordingly, it may be reasonable to assume that subcutaneous immunization induces only subtype-specific immunity since it does not induce sufficient amounts of IgA.

Indeed, we found that the budding and extracellular release of H12N5 virus particles were inhibited in the presence of cross-reactive IgA induced in H9N2 virus-immunized mice (Fig. 6). Thus, the most plausible explanation for the mechanism underlying the inhibitory effects of cross-reactive nonneutralizing IgA antibodies on virus particle budding is that the antibodies crosslink multiple HA molecules expressed on the infected cell surface before initiation of the budding process and, consequently, this intricate cross-linkage via antibodies and HA molecules interferes with the pinch-off of virus particles or release of budded particles. Accordingly, the accumulation of unreleased virus particles on the cell surface was partially observed in electron microscopy, although a quantitative analysis could not be carried out (data not shown).

As mentioned above, the viral budding process is an important target for some antibodies that have protective properties. In addition, IgA antibodies have been shown to bind newly synthesized viral proteins in infected cells and to inhibit virus protein functions intracellularly [43]–[46]. This mechanism may allow cross-reactive anti-HA IgA antibodies to interfere with the maturation of HA (e.g., glycosylation, molecular folding, and proteolytic processing) in infected cells. It might also be possible that IgA antibodies contribute to the antibody-dependent cell-mediated cytotoxicity, which may reduce the production of progeny viruses by readily clearing infected cells. Our data emphasize the idea that the “classical” neutralizing activity is not the only indicator of antibody function contributing to heterosubtypic immunity. A detailed analysis of the precise mechanism by which IgA antibodies interfere with the virus replication may provide new insights into the development of universal mucosal vaccines against multiple subtypes of influenza viruses.
